# Human Natural Killer T Cells Are Heterogeneous in Their Capacity to Reprogram Their Effector Functions

**DOI:** 10.1371/journal.pone.0000050

**Published:** 2006-12-20

**Authors:** Karla A. Eger, Mark S. Sundrud, Alison A. Motsinger, Michelle Tseng, Luc Van Kaer, Derya Unutmaz

**Affiliations:** 1 Department of Microbiology and Immunology, Vanderbilt University School of Medicine Nashville, Tennessee, United States of America; 2 Center for Human Genetics Research, Vanderbilt University School of Medicine Nashville, Tennessee, United States of America; University of California, San Francisco, United States of America

## Abstract

**Background:**

Natural killer T (NKT) cells are a subset of T cells that help potentiate and regulate immune responses. Although human NKT cell subsets with distinct effector functions have been identified, it is unclear whether the effector functions of these subsets are imprinted during development or can be selectively reprogrammed in the periphery.

**Results:**

We found that neonatal NKT cells are predominantly CD4+ and express higher levels of CCR7 and CD62L and lower levels of CD94 and CD161 than adult CD4+ or CD4− NKT cell subsets. Accordingly, neonatal NKT cells were more flexible than adult CD4+ NKT cells in their capacity to acquire Th1- or Th2-like functions upon either cytokine-mediated polarization or ectopic expression of the Th1 or Th2 transcription factors T-bet and GATA-3, respectively. Consistent with their more differentiated phenotype, CD4- NKT cells were predominantly resistant to functional reprogramming and displayed higher cytotoxic function. In contrast to conventional T cells, neither the expression of CXCR3 nor the cytotoxic capacity of neonatal NKT cells could be reprogrammed.

**Conclusions and Significance:**

Together, these results suggest that neonatal CD4+, adult CD4+, and adult CD4− NKT may represent unique states of maturation and that some functions of human NKT cells may be developmentally imprinted, while others are acquired similar to conventional T cell subsets during peripheral maturation and differentiation. Given the potent immuno-regulatory functions of NKT cells, these findings have important implications for the development of novel NKT cell-based therapeutics and vaccines.

## Introduction

NKT cells play important and multifaceted roles in immune regulation, tumor rejection and resistance to a variety of viral, bacterial, and parasitic pathogens through their rapid secretion of immunoregulatory cytokines and potent cytotoxicity [1]–[4]. NKT cells are characterized by a highly conserved T cell receptor (TCR) in both mice and humans [5]–[9], with human NKT cells most reliably identified by the expression of their invariant TCR, consisting of a Vα24-Jα18 chain preferentially paired with a Vβ11 chain [10]–[12]. NKT cells are present at low and highly variable frequencies in human blood (0.01%–1%) but are present at higher numbers in the liver [13]–[15].

In contrast to conventional T cells, NKT cells recognize glycolipid antigens presented by the non-polymorphic MHC class-I like molecule CD1d [5], [16]–[18]. The marine sponge derived glycosphingolipid α-galactosylceramide (α-GalCer) was the first synthetic NKT ligand identified that specifically stimulated NKT cells in the context of CD1d [19]. More recently, NKT cells have been shown to recognize and respond to an endogenous lysosomal glycosphingolipid, isoglobotrihexosylceramide [20]. Several bacterial derived glycolipids have also been recently shown to activate NKT cells [9], [21]–[25].

NKT cells can be phenotypically subdivided into CD4+ and CD4− subsets. A portion of both human and monkey NKT cells also express CD8 [26]–[31]. While human adult NKT cells are comprised of both CD4+ and CD4− subsets, neonatal NKT cells are almost exclusively CD4+ [32]–[34]. Furthermore, CD4+ NKT cells but not CD4− NKT cells have been shown to develop in the human fetal thymus [32], [34]. It remains unclear whether CD4− NKT cells develop from precursor CD4+ NKT cells or as a separate lineage.

Upon activation, NKT cells rapidly secrete large amounts of cytokines, including interferon gamma (IFN-γ) and IL-4. While some functional differences between human CD4+ and CD4− NKT cells have been described [27], [28], [35], it is presently unclear whether NKT cells are solely comprised of subsets that are hardwired to secrete specific cytokine patterns or if some of these subsets can modify their effector functions during peripheral immune activation, analogous to conventional T cells. In contrast to murine NKT cells, which were reported to be resistant to cytokine polarization in vivo [36], upon activation with either environmentally instructed dendritic cells (DCs) or DC1 and DC2 antigen presenting cells (APCs), human NKT cells have been shown to undergo polarization into cells with Th1- or Th2-like cytokine production profiles, termed NKT1 and NKT2 cells [37], [38]. A strong correlation between CD4 expression and the capacity to produce Th2 cytokines has also been reported [27], [28], [35]. Examination of the expression patterns of chemokine receptors and adhesion molecules, which play critical roles in leukocyte trafficking, have revealed further differences between CD4+ and CD4− NKT cells [35].

In the current study, we demonstrate that neonatal CD4+ (nCD4+), adult CD4+ (aCD4+) and adult CD4− (aCD4−) NKT cells are phenotypically distinct in their expression of effector/memory T cell markers and NK cell markers. Consistent with their more naïve phenotype, we also demonstrate that some, but not all, of the effector functions of human nCD4+ NKT cells can be easily modified through either polarizing cytokines or ectopic expression of the master Th1 and Th2 transcription factors (TFs) T-bet and GATA-3, respectively. By comparison, aCD4+ NKT cells were less permissive, whereas aCD4− NKT cells were largely refractory to these polarizing signals. These findings have implications for harnessing and reprogramming the effector functions of human NKT cells in vivo for novel therapeutic and vaccine approaches.

## Results

### Phenotypic characterization of human NKT cell subsets

**Figure 1:**
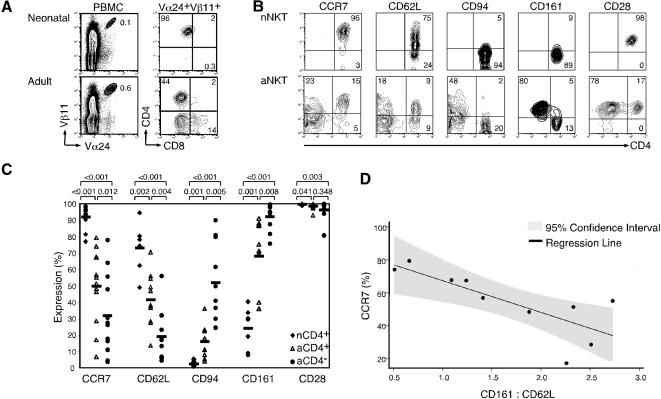
Identification of NKT cell subsets in neonatal and adult blood PBMC. (A) NKT cells in either neonatal or adult PBMC were identified by staining for the invariant TCR using antibodies against Vα24 (FITC) and Vβ11 (unlabelled followed by goat anti-mouse APC). Double positive cells were identified as NKT cells and gated on for further analysis to determine CD4 (PE) and CD8 (biotin followed by strepavidin-PerCP-Cy5.5) expression. (B) NKT cell subsets were identified by staining for Vα24, Vβ11, and CD4 as in 1A and then gated on for further analysis. Expression of the indicated cell surface marker on these subsets was then determined. Combinations of the following antibodies were used, depending on the particular stain: Vα24 (FITC or unlabelled), Vβ11 (unlabelled or PE), CD4 (PE, biotin, or unlabelled), CCR7 (unlabelled), CD62L (PE), CD94 (unlabelled), CD161 (FITC), CD28 (biotin), goat anti-mouse (APC), and strepavidin (PerCP-Cy5.5). One representative donor is shown. (C) Summary of NKT cell phenotype data for all neonatal and adult donors. P values are indicated. For comparison of nCD4+ to either aCD4+ or aCD4− subsets, P values were generated using the Wilcoxon rank sum test. For comparison of aCD4+ to aCD4− subsets, P values were generated using the Wilcoxon signed rank test. (D) For CD4+ NKT cells of adult donors represented in 1C, the ratio of CD161 (% expression) to CD62L (% expression) was generated and compared to CCR7 expression using linear regression with a resulting equation of y = −19.277X+86.676 and p = 0.008.

Human NKT cells can be subdivided into CD4+ and CD4− subsets, which display functionally distinct cytokine secretion profiles [27], [28], [35]. We further characterized the expression profiles of a panel of cell surface markers on resting nCD4+, aCD4+ and aCD4− NKT cells. Neonatal NKT cells were predominantly (>90%) CD4+, whereas adult blood NKT cells were either CD4+ or CD4−, with variable levels of CD8 expression (Figure 1A), which is consistent with previous reports [32], [33]. The chemokine receptor CCR7 and the lymph node homing receptor CD62L, which are specifically expressed on naïve and central memory T cells [39], were expressed highly on nCD4+, moderately on aCD4+, and relatively lower on aCD4− NKT cells (Figures 1B and 1C). In contrast, expression of the NK lineage receptors CD94 and CD161 were mostly absent on nCD4+ T cells but detected at moderate and high levels on aCD4+ and aCD4− NKT cells, respectively (Figures 1B and 1C). Expression of the co-stimulatory molecule CD28 was high on all subsets, but statistically higher on nCD4+ NKT cells compared to either adult NKT cell subsets (Figures 1B and 1C). Because we observed heterogeneous expression of the above markers on aCD4+ NKT cells from different donors, we next determined whether these markers were expressed coordinately on cells from the same donor, such that donors with high levels of CCR7 also expressed high levels of CD62L and low levels of CD161, and vice-versa. Because CD161 and CD62L are inversely expressed (Figure 1C), we calculated the ratio of these values. We found a striking negative correlation (−0.7765) between CCR7 expression and the ratio of CD161 and CD62L. The slope of the associated regression line was statistically significant with a p value = 0.008 (Figure 1D). These results suggest that human nCD4+, aCD4+ and aCD4− NKT cells may represent different stages of maturation and that there is wide disparity in the maturation state of the aCD4+ NKT cell pool, which can be phenotypically subdivided into CCR7^high^CD62L^high^CD161^low^ and CCR7^low^CD62L^low^CD161^high^ populations.

### Ex vivo cytokine polarization of nCD4+, aCD4+ and aCD4− NKT cell subsets

Conventional CD4+ T helper (Th) cells regulate adaptive immune responses by differentiating into Th1 or Th2 effector subsets, which produce either IFN-γ or IL-4, respectively [40]–[42]. Although many factors contribute to the polarization of naïve Th cell effector functions, cytokine signals during activation are critical in this differentiation process. Specifically, IFN-γ and IL-12 are instrumental in directing Th1 differentiation, whereas IL-4 promotes Th2 differentiation [40]–[42]. As naive Th cells differentiate from naïve to effector/memory subsets, heritable chromatin modifications at cytokine gene loci imprint an effector program which results in Th cells that are less capable of reprogramming their effector functions [43], [44]. Therefore, we investigated the extent to which NKT cell subsets that exhibit distinct memory phenotypes could acquire Th1 or Th2-like effector functions when activated in the presence of polarizing cytokine signals.

**Figure 2:**
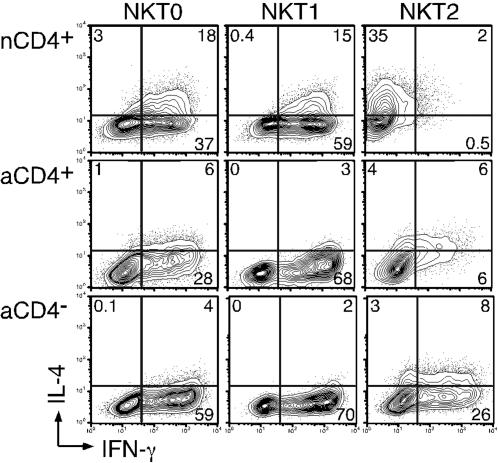
Cytokine polarization of NKT cell subsets. Neonatal or adult NKT cells were purified and activated under conventional Th0 (medium alone; NKT0), Th1 (NKT1), or Th2 (NKT2) polarizing conditions as described in the methods and expanded in IL-2-containing media. These cells were re-stimulated for 6–8 hours using DCs pulsed with α-GalCer (100 ng/ml), or alternatively, anti-CD3 and anti-CD28, in the presence of GolgiStop (BD) and were then intracellularly stained with antibodies against IL-4 (PE) and IFN-γ (APC). NKT cells were also co-stained with anti-Vβ11 (FITC) to exclude contaminating non-NKT cells. For adult NKT cells, CD4 expression was also assessed by cell surface staining (CD4-biotin followed by strepavidin-PerCP-Cy5.5) to differentiate CD4+ from CD4− NKT cells. One representative donor out of 5 is shown.

Purified human neonatal or adult NKT cells were activated by DCs pulsed with α-GalCer in the presence or absence of conventional Th1- (IL-12, neutralizing IL-4 antibody) or Th2- (IL-4, neutralizing IL-12 and IFN-γ antibodies) polarizing conditions, here termed NKT1 and NKT2 respectively. Following activation, NKT cells were expanded for approximately 2.5 weeks and their effector functions were determined. All NKT cell subsets activated with DCs alone (NKT0) expressed high levels of IFN-γ; compared to adult subsets, NKT0 nCD4+ NKT cells also produced relatively higher levels of IL-4 (Figure 2). When neonatal CD4+ NKT cells were activated under NKT1 conditions these cells displayed increased levels of IFN-γ, however expression of IL-4 was not greatly downregulated as compared to NKT0 cells (Figure 2). Under Th2-like polarization signals, nCD4+ NKT cells were easily programmed towards a NKT2 phenotype, as defined by secretion of high levels of IL-4 and low levels of IFN-γ (Figure 2), similar to conventional naïve CD4+ T cells polarized to Th2 cells under the same conditions (Figure S1). In contrast, aCD4− NKT cells, and to a lesser extent aCD4+ NKT cells, were more resistant to NKT2 polarization (Figure 2).

**Figure 3:**
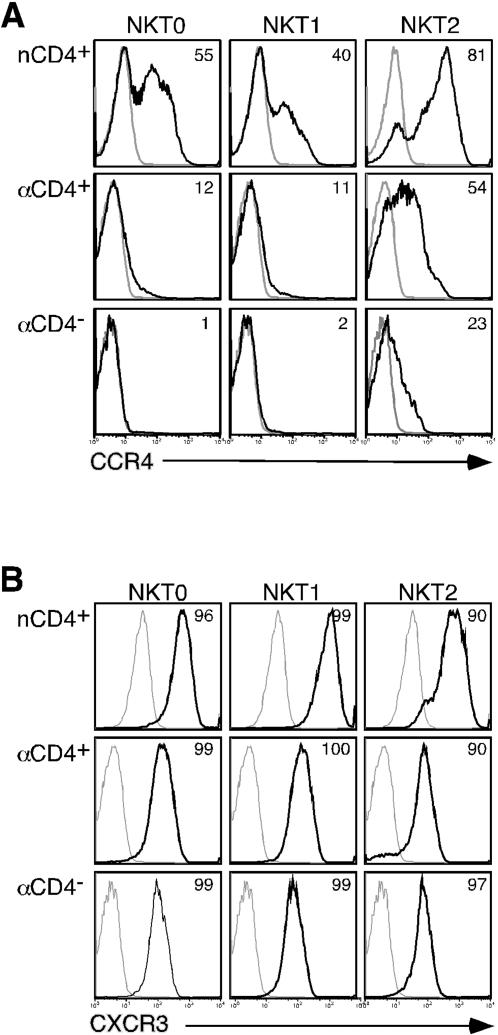
Chemokine receptor expression on polarized NKT cell subsets. One representative neonatal or adult sample of 5 is shown for each. (A) CCR4 expression on polarized NKT cell lines was assayed by staining with anti-CCR4 (unlabelled followed by goat anti-mouse APC) and anti-CD4 (PE) to differentiate between adult CD4+ and CD4− NKT cells and anti-Vβ11 (FITC) to exclude contaminating non-NKT cells. Isotype control is depicted in light gray. (B) CXCR3 (unlabelled followed by goat anti-mouse APC) expression on polarized NKT cell subsets were assayed as in 3A.

Certain chemokine receptors such as CXCR3 and CCR4 are preferentially expressed on Th1 or Th2 cells, respectively [44], [45]. To further examine the extent of NKT cell polarization, we determined the expression levels of these chemokine receptors on cytokine-polarized NKT cell lines. NKT0 nCD4+ NKT cells expressed increased levels of the Th2-associated receptor CCR4 compared to either adult subset (Figure 3A), which is consistent with their higher levels of IL-4 expression (Figure 2). CCR4 expression was strongly induced on nCD4+ NKT cells under NKT2 polarizing conditions, but only moderately so on aCD4+ and even less so on aCD4− NKT cells (Figure 3A). Expression of Th1-specific CXCR3 was highly expressed on all NKT cell subsets and, in stark contrast to conventional Th cells [44], was not substantially modulated by Th2-like cytokine polarization (Figure 3B). Taken together, these results suggest that nCD4+, and to a lesser extent aCD4+ NKT cells, have an enhanced capacity to modify their effector functions as compared with their CD4− counterparts. However, in contrast to conventional T cells, NKT cells also display some pre-programmed characteristics, which could not be modulated by cytokine polarizing signals.

### Reprogramming NKT cell subset effector functions by ectopic expression of the transcription factors GATA-3 and T-bet

**Figure 4:**
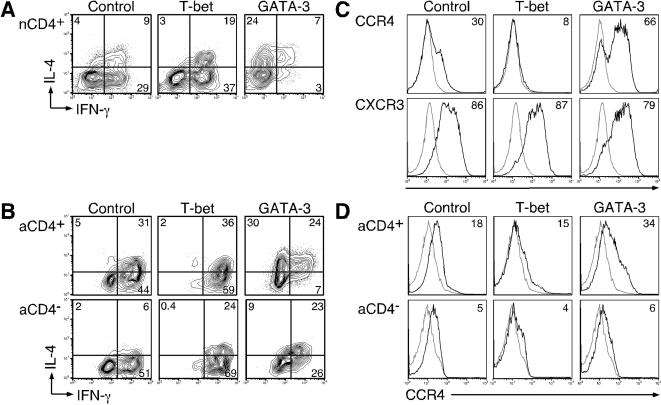
Functional profile of NKT cell subsets ectopically expressing T-bet or GATA-3. NKT cells were activated under non-polarizing conditions as in Figure 2, transduced at the time of activation with either the control lentiviral vector or vectors expressing T-bet or GATA3, and expanded in IL-2-containing media. One representative donor out of 3 is shown for each. (A) Intracellular stain of nCD4+ NKT cells was performed as in Figure 2 and cells were co-stained with anti-mCD24 (FITC) to identify transduced cells. (B) Intracellular cytokine stains of aCD4+ or aCD4− NKT cells were performed as in 4A. CD4 expression was determined by co-staining with anti-CD4 (biotin followed with strepavidin PerCP-Cy 5.5). (C) CCR4 or CXCR3 expression on T-bet- or GATA-3-transduced nNKT cells was assayed as in Figures 3A and 3B, and cells were co-stained with anti-mCD24 (FITC) as a marker for transduction. (D) CCR4 expression of T-bet or GATA-3-transduced aCD4+ or aCD4− NKT cell was examined as in 4C, and cells were stained with anti-CD4 (PE) to differentiate between CD4+ and CD4− NKT cells.

In conventional Th cells, Th1 and Th2 differentiation are directed by the master transcription factors (TFs) T-bet and GATA-3, respectively [46]–[48]. Whereas T-bet or GATA-3 are sufficient to program Th1 or Th2 effector function when ectopically expressed in conventional naïve Th cells, these same TFs are insufficient to reprogram the established effector functions of memory Th cell subsets [44]. Therefore, we asked whether bypassing cytokine polarization signals by ectopically expressing T-bet or GATA-3 could overcome the resistance of adult NKT cells to effector function reprogramming. Accordingly, NKT cells were purified as before, activated using DCs pulsed with α−GalCer and simultaneously transduced with a lentiviral vector expressing either T-bet or GATA-3, in conjunction with a reporter cell surface marker, murine CD24 (mCD24). An empty vector expressing mCD24 alone was used as a negative control. Transduced NKT cells were then expanded and assayed for cytokine production and chemokine receptor expression as before, using mCD24 as a marker for transduction. Similar to cytokine polarization, T-bet or GATA-3 expression in nCD4+ NKT cells was sufficient to drive NKT1 or NKT2 polarization, respectively (Figures 4A and 4C). As with the cytokine polarization experiments, ectopic expression of either of these TFs did not modulate CXCR3 expression on nCD4+ cells (Figure 4C). These results suggests that T-bet or GATA-3 can partially function to program the effector function of nCD4+ NKT cells. Ectopic expression of these TFs in either aCD4+ or aCD4− NKT cell subsets was unable to overcome the partial resistance of these subsets to effector function reprogramming by cytokine signals (Figures 4B and 4D). Taken together, these results suggest that, similar to conventional effector/memory CD4+ Th subsets [44], bypassing cytokine receptor signaling does not notably overcome the resistance of aCD4− NKT cells to effector function reprogramming.

### Differential cytotoxic function of human NKT cell subsets

**Figure 5:**
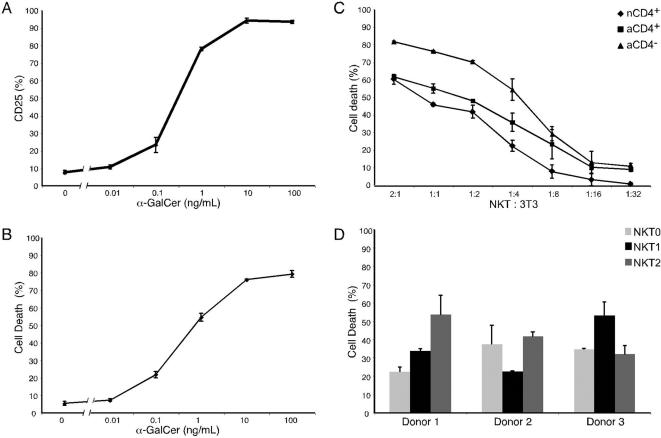
Cytotoxic capability of NKT cell subsets. Experiments were performed in triplicate per donor; standard deviation is depicted. For A, B, C, and D, one representative donor out of three independent experiments is shown. (A) NKT cells were added at an Effector:Target (NKT cell: 3T3 cell) ratio of 1∶1 to adherent 3T3 cells in the presence of different α-GalCer concentrations. After 18–20 hours, NKT cells were collected and stained with anti-CD25 (unlabelled followed by goat anti-mouse APC) to determine activation. (B) As in A, NKT cells at an NKT:3T3 of 1∶1 were activated in the presence of varying concentrations of α-GalCer. After 18–20 hours, cells were collected by trypsinization to remove any 3T3 cells that were still adherent and target 3T3 cells were stained with propidium iodide to determine their viability. (C) α-GalCer was added at a concentration of 100 ng/ml to adherent 3T3 cells. NKT cell subsets were then added at different NKT:3T3 ratios. Viability of 3T3 cells after an 18–20 hour incubation period was determined as in 5B. (D) Polarized nCD4+ NKT cells were generated as in Figure 2. The cytotoxic capacity of these NKT0, NKT1, and NKT2 lines was assayed as in Figure 5C. One NKT:3T3 ratio for each donor is shown.

One of the characteristics of NKT cells is their cytotoxic function [49]. We therefore asked whether NKT cell subsets differed in their cytotoxic potential and whether this activity could be regulated by cytokine polarization. For these experiments, we developed a cytotoxicity assay using a murine fibroblast line (NIH 3T3 cells) which endogenously expresses CD1d and can present α-GalCer to activate human NKT cells in a dose-dependent fashion, as measured by up-regulation of the IL-2 receptor α chain, CD25 (Figure 5A). Further, upon TCR stimulation, NKT cells can kill target 3T3 cells, also in an α-GalCer dose-dependent fashion (Figure 5B). Using this assay, we tested the cytotoxic potential of in vitro generated nCD4+, aCD4+ and aCD4− NKT cell lines. We found that aCD4− NKT cells had higher cytotoxic activity against α-GalCer presenting target 3T3 cells compared to either nCD4+ or aCD4+ NKT cells at a variety of effector to target ratios (Figure 5C).

Because conventional Th1 CD4+ Th cell lines can exhibit higher cytotoxicity relative to Th2 cells upon activation (Figure S2) [50], we next asked whether NKT1 and NKT2 cells displayed differences in their cytotoxic effector functions. For this experiment we used nCD4+ NKT cells, since these could be much more readily differentiated into NKT1 or NKT2 type cells (Figures 2 and 3A). In contrast to conventional CD4+ Th1 and Th2 subsets activated with SEB and tested under these conditions (Figure S2), nCD4+ NKT1 and NKT2 cells were similar in their cytotoxic capacity (Figure 5D). These results suggest that, unlike cytokine production and similar to CXCR3 expression, the cytotoxic function of NKT cells appears to be developmentally imprinted.

## Discussion

Here, we have demonstrated that NKT cells from neonates are phenotypically and functionally distinct from adult NKT cell subsets. In comparison to aCD4+ NKT cells, nCD4+ NKT cells exhibit: 1) higher expression of the differentiation markers CCR7 and CD62L, 2) lower expression of the NK cell markers CD161 and CD94, and 3) are more amenable to effector function reprogramming. We also found wide heterogeneity within the aCD4+ NKT cell subset for expression of memory markers. These findings suggest that the pool of CD4+ NKT cells in adult peripheral blood represents a more heterogeneous mixture of cells with respect to their phenotype and stage of maturation. This premise would predict that adult NKT cells are not recent thymic emigrants, but have undergone some form of peripheral maturation. Several lines of evidence are consistent with our hypothesis. In one study, Vα24+CD4+ NKT cell progenitors were observed to be present in the early fetal thymus but rare in the postnatal thymus, again suggesting the possibility that adult CD4+ NKT cells are likely to be peripherally expanded cells rather than recent thymic emigrants [34]; other studies also reported low levels of NKT cells in the postnatal thymus [32], [33]. Analysis of T cell receptor excision circles in NKT cells suggested that aCD4+ NKT cells found in the periphery were a result of both thymic output and peripheral expansion [32]. Despite expression of the memory marker CD45RO, resting neonatal NKT cells were also reported to be functionally immature as defined by their inability to produce IL-4 or IFN-γ upon primary activation and only acquired their effector functions after cell division, implying that in vivo activation and peripheral expansion are necessary to attain functional maturation [32], [51].

Several lines of evidence presented here also clearly suggest that aCD4− NKT cells are more differentiated compared to CD4+ subsets: 1) aCD4− NKT cells displayed lower expression levels of CD62L and CCR7 and increased expression levels of CD94 and CD161, 2) aCD4− NKT cells were predominantly resistant to reprogramming either via polarizing cytokine signals or ectopic expression of T-bet or GATA-3, and 3) aCD4− NKT cells also displayed greater cytotoxic activity compared to either nCD4+ or aCD4+ NKT cells. Based on these findings, we hypothesize that CD4 is a differentiation marker for human NKT cells, such that CD4− NKT cells may possibly represent a product of precursor CD4+ NKT cells which have undergone peripheral maturation. This hypothesis is also supported by the findings that aCD4+ NKT cells contain a higher level of T cell receptor excision circles than aCD4− NKT cells [32] and that CD4− NKT cells are absent in neonates and only begin to accumulate after about 6 months of age [32]–[34].

Conventional Th cells progressively lose the capacity to modify their effector functions as they differentiate from naïve cells to central and effector memory cells [43], [44]. Therefore the flexibility of nCD4+, aCD4+ and aCD4− NKT cell subsets to reprogram effector functions could be a reflection of their differentiation state, as defined by the expression of memory T cell markers and NK cell lineage receptors, as well as their cytotoxic capacity. We speculate that the phenotypic and functional profiles of neonatal CD4+ and adult CD4− NKT cells are analogous to conventional naïve and memory Th cell profiles [39], [43], [44]. However, in contrast to conventional Th cells, several NKT cell effector functions (such as cytotoxity and CXCR3 expression), which can be modulated in conventional T cells, were not responsive to polarizing cytokine signals or forced expression of TFs. We also observed that nCD4+ cells had higher expression levels of IL-4 under NKT1 polarizing conditions compared to conventional Th1 cells, which rapidly silence the IL-4 locus. However, because the aCD4− subset has reduced expression of IL-4 and CCR4 in non-polarizing conditions [27], [28], [35] (Figures 2 and 3A), as well as increased cytotoxicity (Figure 5C), it is conceivable that these functions are modified in vivo as NKT cells are activated by antigens and/or cytokines such as IL-12, which has been speculated to contribute to the peripheral maturation of NKT cells [52]. However, the importance of factors such as co-stimulation, cytokine signals present at the time of activation, strength and length of signal and the nature of the antigen, whether endogenous or foreign, in the differentiation and maturation process of NKT cells remains to be elucidated.

The functional differences between conventional Th and NKT cells described in these studies could potentially result from distinct, lineage-specific transcriptional regulatory mechanisms, or alternatively, may reflect unique developmentally imprinted genetic programs. As such, because the positive selection of NKT cells is mediated by double-positive thymocytes rather than cortical epithelial cells [53], it is possible that the unique developmental program of NKT cells may result in qualitatively different signals that impact subsequent functional programs. Indeed, NKT cell development differs from conventional T cells in the required expression of several transcription and signaling molecules, including T-bet, NF-κB and PKC-θ[54]–[58], thus suggesting unique developmental signals.

While we have shown here that certain functions of NKT cells are likely to be developmentally imprinted, others can be readily modulated, at least in the CD4+ NKT cell subset. It is not clear how NKT cells would acquire different functional profiles in vivo. Although murine NKT cells were reported to be resistant to cytokine polarization in vivo [36], direct ex-vivo cytokine mediated polarization was not assessed and in various other experimental systems evidence suggests that the nature of the antigen [17], [59]–[62], the type of antigen presenting cell [37], [38], [63], and the presence or absence of co-stimulatory signals [64], [65] could all play a role in modulating the functional and phenotypic profiles of NKT cells. For example, distinct structural analogs of α−GalCer can elicit different cytokine secretion profiles by NKT cells [17], [59]–[62]. While both human NKT cell subsets produce IFN-γ, CD4+ NKT cells produce higher levels of Th2 cytokines than CD4− NKT cells, suggesting that NKT cell effector function may also be regulated through the selective recruitment and activation of these subsets [27], [28], [35]. Furthermore, it was recently demonstrated that murine NKT cells from different organs may mediate distinct functions [66], raising the possibility that naïve-like NKT cell subsets may be recruited to different organs where they can undergo divergent effector function programming into distinct effector subsets. Indeed, similar mechanisms appear to be in place for conventional Th cells, whereby specialized, tissue-specific APCs can fine-tune T cell effector function [67].

Dysregulation of NKT cell function has been linked to a variety of immune deficiencies and pathologies. In comparison to normal healthy donors, which possess very low levels of NKT cells in the lung, on average a striking 60% of CD3+ cells in the lungs of bronchial asthma patients were NKT cells, with greater than 90% representing CD4+ NKT cells that expressed IL-4 but little IFN-γ [68]. Other examples of the role of NKT cells in autoimmunity, tumor biology and infection have also been reviewed [4], [9]. Our findings highlight the possibility that therapeutic manipulation of NKT cells to improve immune responses against autoimmune or infectious diseases may be most optimally achieved by targeting specific subsets rather than the entire NKT cell pool. While CD4+ NKT cells are amenable to reprogramming, CD4− NKT cells are functionally static. Conceivably, some autoimmune conditions may be circumvented by activation conditions that preferentially target CD4+ NKT cells and favor the development of strong Th2 responses. Also, the potent anti-tumor or anti-viral activity of NKT cells may be most beneficial under activation conditions that preferentially favor the development of either NKT1 or CD4− NKT cells, which can potently mediate the downstream activation of NK cells and CD8+ cytotoxic T lymphocytes, further amplifying anti-tumor or viral activity. Of note and in contrast to conventional T cells, we were unable to modulate the direct cytotoxic activity of NKT cells. As a better understanding of the mechanisms that regulate NKT cell function is achieved and as novel ligands that can specifically modulate these functions are generated, it may be possible to specifically boost these types of responses in vivo.

In conclusion, our results reveal both imprinted and highly flexible differentiation and effector programs of neonatal NKT cells, whose flexibility progressively diminish in adult NKT cells as they acquire a more differentiated phenotype. These findings suggest that CD4+ and CD4− NKT cells may represent unique differentiation states. While we cannot exclude the possibility that CD4− NKT cells may develop as a separate lineage, our studies in conjunction with previous reports [32], [34], support a linear model of human NKT cell differentiation. Because NKT cells serve as an important link between the innate and adaptive immune system, our results have important implications for the design of novel therapeutic interventions aimed at harnessing the immunoregulatory potential of NKT cells.

## Materials and Methods

### Purification and preparation of primary human T cell subsets and DCs

PBMC were isolated from either neonatal cord blood or peripheral adult blood from healthy individuals by Ficoll (Pharmacia) density centrifugation. NKT cells were enriched from adult PBMC or neonatal CD4+ T cells by staining with Vα24 antibody, followed by goat anti-mouse magnetic beads (Miltenyi Biotec), and positively sorted by magnetic cell sorting using AutoMACS (Miltenyi Biotec) as described [30]. CD4+ conventional T cells (Th cells) were purified from PBMC using Dynabead anti-CD4 antibodies and magnetic separation, as previously described [44]. Monocyte-derived DCs were prepared as previously described [69], [70]. DCs were matured by stimulation with 10 ng/ml LPS (Sigma) one day prior to T cell activation. All subjects provided written informed consent that was approved by the Vanderbilt Institutional Review Board.

### T cell activation and differentiation

Purified Vα24+ NKT cells were stimulated with α-GalCer (100 ng/mL, Kirin Brewery Co., Gunma, Japan) using mature DCs. CD4+ Th cells were activated by DCs using Staphylococcus enterotoxin B (SEB, 20 ng/mL, Sigma). For cytokine polarization, cells were activated in the presence or absence of polarizing cytokine conditions as previously described for conventional CD4+ Th cells [44] under the following conditions: human rhIL-12 (30 ng/ml) and neutralizing anti-IL-4 antibody (0.5 µg/mL) for Th1 conditions and human rhIL-4 (50 ng/mL) and neutralizing anti-IFN-γ (2.5 µg/ml) and anti-IL-12 (2.5 µg/ml) antibodies for Th2 conditions (all from R&D Systems) [44]. Cells were expanded in rhIL-2 containing media. The culture media used in all experiments was as described [70]. For purification of aCD4+ or aCD4− NKT cells, from expanded adult NKT cell lines, cells were stained with Vβ11 (Immunotech) to exclude any contaminating non-NKT cells and CD4 (BD Pharmingen); aCD4+ and aCD4− NKT cells were sorted using flow cytometry (FACS Aria, BD Pharmingen).

### Virus production and infections

Vesicular stomatitis virus glycoprotein (VSV-G)-pseudotyped lentiviruses were generated as previously described [70]. Briefly, HEK-293T cells were transfected with lentiviral vectors and VSV-G envelope plasmids. Supernatants were collected 48 h post-transfection and filtered through 0.45 µM filters. Construction and use of lentiviral vectors expressing a marker gene (murine CD24, mCD24) and either GATA-3 or T-bet have also been described [44], [70]. For transduction experiments, NKT and conventional CD4+ Th cells were infected at the time of activation at a multiplicity of infection of 3–8.

### Staining and FACS analysis

Cells were stained with the relevant antibody and analyzed using a flow cytometer (FACSCalibur) as described [44]. Analysis was performed using FlowJo software (Tree Star). The following anti-human antibodies were used for staining: Vα24, Vβ11 (Immunotech); CD4, CD8, CD28, CD94, CD161, CD25, CCR4, CXCR3, CD62L (all from BD Pharmingen) and CCR7 (R&D Systems). In some experiments mCD24 antibody (BD Pharmingen) was used as marker for lentivirus-transduced T cells. Secondary goat anti-mouse antibody and streptavidin conjugates were from BD Pharmingen and pure mouse IgG1 was purchased from Caltag.

### Cytokine expression assays

For intracellular cytokine analysis, NKT and CD4+ Th cells were stimulated with α−GalCer (100 ng/ml) or SEB (20 ng/ml) pulsed DCs, respectively, or alternatively by cross-linking the TCR with antibodies against CD3 (OKT3, American Type Culture Collection (ATCC)) and CD28 (BD Biosciences), in the presence of 0.67 µl/ml GolgiStop (BD Pharmingen) and incubated for 6–8 hours at 37°C. This was followed by cell surface staining for CD4 to identify cells from either the CD4+ or CD4− lineage and anti-Vβ11 to exclude any contaminating non-NKT cells. Lentiviral transduction was monitored using anti-mCD24. Subsequently, cells were fixed and permeabilized using a commercial kit (BD Pharmingen) according to the manufacturer's instructions. Cells were then stained with the following anti-human cytokine antibodies: IFN-γ and IL-4 (both from BD Pharmingen).

### Cytotoxicity Assay

Murine fibroblast cells (NIH-3T3) expressing endogenous murine CD1d were seeded in a 96 well plate at 8,000 cells per well and incubated for 1–3 h to adhere. To activate NKT cells, α-GalCer (between 0.01–100 ng/ml) was added to these cultures. For CD4+ Th cell activations, 3T3 cells ectopically expressing the human class II molecule HLA-DR were pulsed with SEB (20 ng/mL). Effector cells (NKT or Th) were then added at various effector to target ratios (2∶1 to 1∶32). After 18–20 hours, cells were trypsinized, collected and stained with propidium iodide (PI, 10 µg/ml, Sigma) or anti-CD25 and analyzed by flow cytometry. For experiments comparing different NKT or Th lines, a negative control was used for normalization between samples. 3T3 cells were distinguished from NKT or CD4+ Th cells by their distinctive larger forward and side scatter profiles.

### Statistical Analysis

Statistical analyses were performed using STATA version 9.0. In all cases, a p-value<0.05 was considered statistically significant. Due to the non-normal nature of the distributions, non-parametric tests were used for all comparisons in order to avoid spurious results. Linear regression was used to characterize the relationship between CCR7 expression and the ratio of CD161∶CD62L expression in aCD4+ NKT cells. A Kruskal-Wallis test was used for the initial comparisons of the distribution of cell surface marker expression profiles of nCD4+, aCD4+, and aCD4− NKT cell subsets, followed by pair-wise comparison tests. For non-matched comparisons, the Wilcoxon rank-sum test was used, and for matched comparisons, the Wilcoxon's signed rank test was performed. It is important to note that the p-values presented are uncorrected for the multiple tests performed in the pair-wise comparisons, yet even with a highly conservative Bonferroni correction, all results except one are still statistically significant.

## Supporting Information

Figure S1 Cytokine polarization of CB CD4+ Th cells. CB CD4+ T helper cells were activated under Th0- Th1- or Th2-polarizing conditions as described in the methods and expanded in IL-2-containing media. These cells were re-stimulated using DCs pulsed with SEB (20 ng/ml) or anti-CD3 and anti-CD28 in the presence of GolgiStop (6-8 hrs) and subsequently stained intracellularly with anti-IL-4 (PE) and anti-IFN-γ (APC).
(0.08 MB TIF)

Figure S2 Cytotoxic capacity of polarized neonatal Th cells. Polarized Th1 or Th2 neonatal lines were generated as in Figure S1. The cytotoxic capacity upon activation at different Th:3T3 ratios of these lines was assayed as in Figure 5B using 20ng/ml SEB for each. Experiments were performed in triplicate per donor; standard deviation is depicted. One representative donor out of three is shown.
(0.08 MB TIF)
