# Supplementary figures and images for: Human Natural Killer T Cells Are Heterogeneous in Their Capacity to Reprogram Their Effector Functions
Source: PLoS One. 2006 Dec 20;1(1):e50. doi: 10.1371/journal.pone.0000050 (PMC1762372; doi:10.1371/journal.pone.0000050)

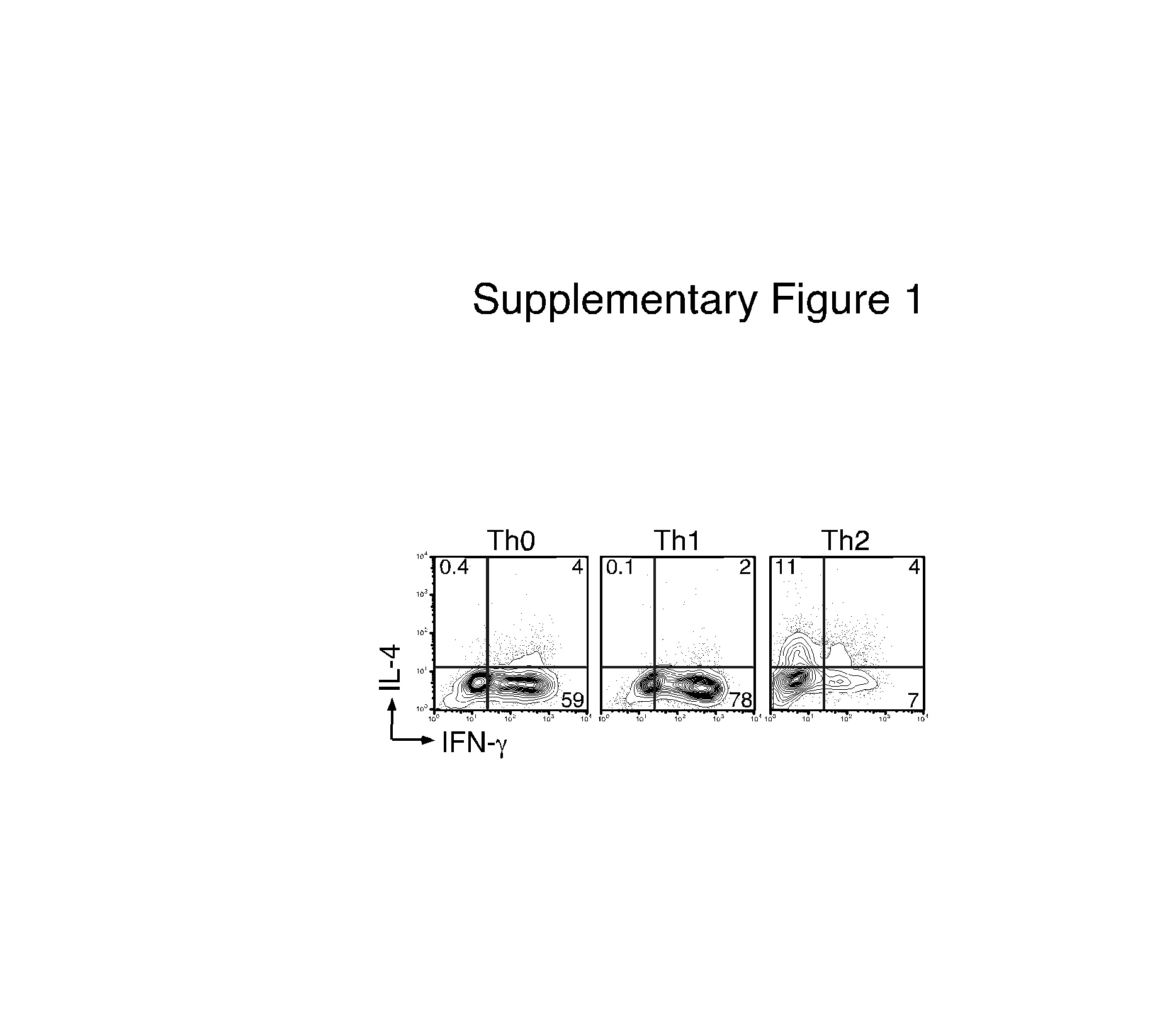

Supplement: Figure S1 — Cytokine polarization of CB CD4+ Th cells. CB CD4+ T helper cells were activated under Th0- Th1- or Th2-polarizing conditions as described in the methods and expanded in IL-2-containing media. These cells were re-stimulated using DCs pulsed with SEB (20 ng/ml) or anti-CD3 and anti-CD28 in the presence of GolgiStop (6-8 hrs) and subsequently stained intracellularly with anti-IL-4 (PE) and anti-IFN-γ (APC). (0.08 MB TIF) [file pone.0000050.s001.tif]

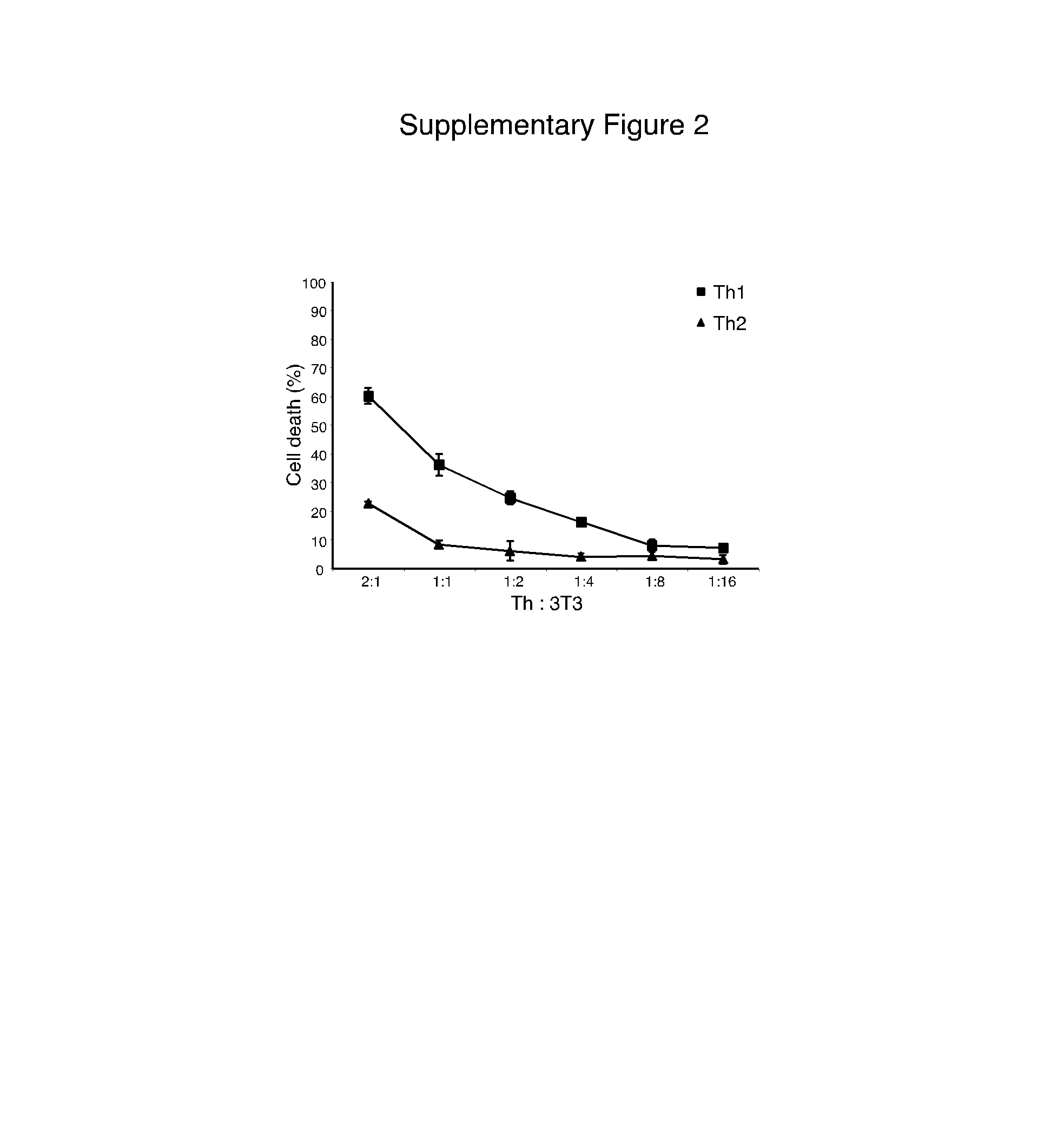

Supplement: Figure S2 — Cytotoxic capacity of polarized neonatal Th cells. Polarized Th1 or Th2 neonatal lines were generated as in Figure S1. The cytotoxic capacity upon activation at different Th:3T3 ratios of these lines was assayed as in Figure 5B using 20ng/ml SEB for each. Experiments were performed in triplicate per donor; standard deviation is depicted. One representative donor out of three is shown. (0.08 MB TIF) [file pone.0000050.s002.tif]
